
JOURNAL INFORMATION
==============================
NLM Title Abbreviation: Atlanta Med Surg J (1884)
NLM Journal ID: 101251623

Atlanta Medical and Surgical Journal (1884)

Publisher: Biomedical Journal Digitization Project

ARTICLE INFORMATION
==============================
PMCID: PMC9199289
PMID: 35829588
Article version: 1
Subjects: Editorial

Does It Pay a Physician to Study?

Print publication date: 1898 Dec
Volume: 15
Volume Series: New Ser
Issue: 10
First page: 687
Last page: 692
License: This work is free of known copyright restrictions. For more information, please see PMC Back Issue Digitization.
License URL: https://creativecommons.org/publicdomain/mark/1.0/

==============================

lead to a serious setback to the scientific investigation of the disorder. Dr. Müller was considered quite an authority on the plague. As a member of a committee consisting of himself, Dr. Albrecht, Dr. Ghon, and Dr. Poech, appointed by the Imperial-Royal Academy of Science, he proceeded in 1897 to Bombay and other points in India for the purpose of studying the plague. The committee returned some months ago to Vienna, and the report of its members has been noted in these columns."

---

### DOES IT PAY A PHYSICIAN TO STUDY?

THERE are a great many *ups* and *downs* in a physician's life, and to them, we have no doubt, the *downs* seem very much in preponderance. It is really discouraging to the close student of medicine to find often that his ability which must necessarily come as a student is not recognized by the public, but the "hail-fellow-well-met," is the one holding the highest favor in the public eye. A recent editorial in the *Colorado Medical Journal* has certainly put the truth of the whole affair in a few very terse sentences. It says:

"This is a simple question which apparently has but one answer, but a friend of ours recently took the negative side of the question and this is about what he said: 'No, it doesn't pay. The people don't want a physician who knows things. They want a man who belongs to their church, to their lodge, a hail-fellow-well-met. He can make a show of learning, can write a prescription with a flourish, can assume an air of learning, and that is all that is needed. The man who puts in his time at his books, at his microscope, in his laboratory, gets left, and the physician who goes past his books and mixes with the people, is the man who coins the 'wherewith.'

"Occasionally this successful man needs the consultation-services of the student, but just as often it goes to the man who has a like successful practice. It is dollars to doughnuts that the physician with a great big practice makes but few diagnoses and treats symptoms only. Why it can't pay to study much. Whom can it pay? A side-light on the same idea came to me the other day. I have been for years using my microscope and with many a case it has given me some severe shocks. It has in fact discouraged me with certain cases and the patient has felt my discouragement. I find that I am using my microscope less than I formerly did. To find an abundance of tubercle bacilli in sputum, while the patient seems in the best of health and spirits, makes me down in the mouth and the patient finds a more hopeful doctor. Casts in the urine always frighten me, and I warn the patient until he gets discouraged. No, it doesn't pay to study and work in this profession. Specialists are now so numerous and consultations are so wanted by the people, that I don't need to know much about the nervous system, nor about the alimentary canal, nor about the ear, nose or throat, nor about the kidneys, nor the heart or chest; and in fact, unless I go to the country to practice, it is a waste of time for me to learn new facts about these things. If I get a section of a tumor, I am expected to turn it over to a pathologist; if I secure some peculiar fluid, I am expected to send it to this man or that man for analysis, and thus it goes, and if I study on these things, I only confirm the reports which I receive. No, it doesn't pay. I can get ahead better by reading novels and going to church socials and balls.'

"Our friend got up, lit a cigarette, and said: 'Good-night, I am due now at an executive committee meeting of 'The Royal Peacocks of the Purple Shadow.'"

---

THE Tri-State Medical Association, comprising the States of Alabama, Georgia and Tennessee, held its annual meeting in Birmingham, Ala., on October 25th, 26th and 27th. The meeting was well attended, the largest representation being from

Alabama, as was to be naturally expected. Dr. George S. Brown, of Birmingham, as Chairman of the Committee of Arrangements, and one of the most enthusiastic members of the whole body, called the meeting to order.

He was followed by Dr. A. T. Henly, President of the Jefferson County Medical Society, who delivered a very earnest address of welcome, at the conclusion of which Dr. J. A. Goggans, of Alexander City, the President of the Association, called the meeting formally to order.

The Secretary's report by Dr. Frank T. Smith showed the Association to be in a most flourishing condition, and after the reading of this the regular program was started.

The attendance on the first day was rather small, but by the second, members and visitors had swelled the numbers present to a considerable degree.

There were a number of excellent papers read during the meeting, and altogether the progress was most successful. The papers elicited excellent discussions. The social features of the meeting added much entertainment and pleasure to the whole affair. On the first evening Drs. Davis, of Birmingham, gave a delightful dinner to a number of the visiting physicians. On the second day a special excursion was given to Pratt City, the physicians being the guests of the Tennessee Coal, Iron and Railroad Company and Dr. R. M. Cunningham, of that city. A view was thus afforded of the immense new steel industry in this portion of Alabama. The trip was greatly enjoyed. At night a general reception was held at the Commercial Club, tendered to the visitors by the Jefferson County Medical Society.

The new officers elected were as follows: President, Dr. George A. Baxter, Chattanooga, Tenn.; First Vice-President, Dr. M. C. McGammon, Nashville, Tenn.; Second Vice-President, Dr. W. D. Travis, Covington, Ga.; Third Vice-President, Dr. George S.

Brown, Birmingham, Ala.; Secretary, Dr. Frank T. Smith, Chattanooga, Tenn.; Treasurer, Dr. Cooper Holtzclaw, Chattanooga, Tenn.

The next place of meeting will be Chattanooga.

---

THE Investigating Committee, appointed by the President of the United States to look into the medical matters incident to the recent Spanish-American war, is now making a tour of the various hospitals and camps, and trying to find out whether there was mismanagement of the medical department during the war, and if so, where the blame should be put. The men composing the committee are men of integrity, and will no doubt try to do their duty, but we cannot see the necessity for the existence of such a committee. The Lord knows the poor people are being taxed to death for the furtherance of the wishes of jingo politicians, yellow journalism, and the administration in office, who forced upon the American people the war which is now assuming the shape of one of conquest, with no limitation as to what the dear people will suffer who have absolutely no voice in the matter. The expenses during the war and the management of the medical department were certainly heavy enough without giving a few men a pleasure trip at the expense of the dear people, and the whole affair to wind up with a reception and an ignominious fiasco. There is no one who believes for a moment that there will anything result from this Investigation Committee's work, for they are too sensible of the administration's feelings to wound such in the slightest degree.

The future condition of this country is indeed an alarming one to our mind, since the dear people must suffer and be taxed to death in order to further the interests of those in power, and those "standing in close touch with the administration." But then why should such views be expressed? for there seems to be no remedy, and the "toiling millions" must grovel for a living, and let the "rich grow richer."

The United States is now made up of every nationality in the world, and with such an admixture of races who can doubt but that this country must be the seat of continual turmoil and strife. Not satisfied with trying to govern this heterogeneous mass already within our present territorial limits, the government, aided by "yellow journalism" and jingo politicians, are seeking to extend our territory in order to create still greater troubles and dissensions, to allow the dear people to pay still more for what they get, and by increased taxation to let them aid the extension of rich men's monopolies to El Dorados purchased for them at the expense of American blood. Shades of our fathers, come and help us!

---

AUSTIN, TEXAS, is to be congratulated upon the thoroughly scientific and sanitary spirit of its city council. This body has just passed a measure which could well be adopted by other municipal governments. We give the ordinance as recently passed.

The following ordinance was introduced by Mr. Jeff. McLemore, and was passed by the city council of the city of Austin, October 3, 1898:

"An ordinance regulating the sale and use of cocaine in the city of Austin, and prescribing penalties.

"Be it ordained by the city council of the city of Austin:

SECTION 1. That it shall be unlawful for any person in the city of Austin to sell or give cocaine to another except upon the prescription of a regularly practicing physician, certifying that he has examined such person professionally, and that he finds such person to be in such physical condition as to be in need of cocaine as a medicine.

"SECTION 2. That it shall be unlawful for any person to make more than one sale or gift of cocaine upon each prescription or pay any physician to give a prescription to another without making a thorough examination of such person.

"SECTION 3. That the violation of any of the provisions of this ordinance is made an offense, and for each violation the person offending shall be fined in any sum not less than $10 nor more than $100.

"SECTION 4. That the provisions of this ordinance shall not apply to the sale or gift of cocaine to a regularly practicing physician or dentist, or to the administration of the same by such physician or dentist personally."

---

A NEW army hospital, the largest under the control of the government, is to be constructed of Georgia pine, at Savannah, Ga., at a cost of $150,000. There will be 149 buildings, all designed and constructed with a view to permanency. The ground plan of buildings will be rectangular, with covered ways connecting all of the buildings with each other, and with the administration-building in the center. Each of the four sides of the quadrangle will contain a ward with a capacity of 250 beds. One ward will be devoted to surgical cases, and an enclosed passage-way will connect it with a modern operating-room. In addition to the general wards there will be private rooms for the accommodation of invalid officers. An unlimited supply of artesian water will be provided. Coal base-burners will supply heat. An ice factory is situated within fifty yards of the site, and the cost of ice is inconsiderable. The officers' quarters will be of two stories. The dormitories for the nurses and the hospital-corps will occupy two buildings. The chief surgeon will have a private residence.
